# The Circadian Syndrome Predicts Lower Urinary Tract Symptoms Suggestive of Benign Prostatic Hyperplasia Better Than Metabolic Syndrome in Aging Males: A 4-Year Follow-Up Study

**DOI:** 10.3389/fmed.2021.715830

**Published:** 2021-09-21

**Authors:** Yang Xiong, Fuxun Zhang, Changjing Wu, Yangchang Zhang, Xiaoyingzi Huang, Feng Qin, Jiuhong Yuan

**Affiliations:** ^1^Andrology Laboratory, West China Hospital, Sichuan University, Chengdu, China; ^2^Department of Urology, West China Hospital, Sichuan University, Chengdu, China; ^3^Department of Epidemiology and Health Statistics, School of Public Health and Management, Chongqing Medical University, Chongqing, China

**Keywords:** aging males, benign prostatic hyperplasia, circadian syndrome, lower urinary tract symptoms, metabolic syndrome

## Abstract

**Background:** The prevalence of lower urinary tract symptoms (LUTS) suggestive of benign prostate hyperplasia (BPH) increases in men. Although several risk factors, including metabolic syndrome (MetS) and depression, were identified, the underlying etiological factor remains unclear. Recently, circadian syndrome (CircS) was proposed as a novel risk cluster based on MetS. To compare the predictive power of the CircS and MetS for LUTS/BPH, this study was performed.

**Materials and Methods:** In the baseline survey, 4,390 men older than 40 years from the China Health and Retirement Longitudinal Study were enrolled. Of them, 3,658 men were followed in the 2015 survey. Logistic regression was adopted to examine the relationships between CircS, MetS, and LUTS/BPH. To further verify the association, propensity score matching was used for sensitivity analyses. Moreover, the participants who had LUTS/BPH at the baseline were excluded to test the longitudinal relationships between CircS, MetS, and LUTS/BPH. In addition, we employed the receiver operating characteristic (ROC) curve analysis to compare the predictive power using the number of components of CircS and MetS. The DeLong test was used to test the disparities of area under the curves (AUCs).

**Results:** The prevalence of CircS and MetS in aging men was 30.23 and 38.36%, respectively. The odds ratios for prevalent LUTS/BPH were 1.61 (95% CI = 1.29–2.00, *P* < 0.001) and 1.34 (95% CI = 1.08–1.66, *P* < 0.01), respectively, in aging men. This increased risk was also observed in incident LUTS/BPH. The prevalence of LUTS/BPH in normal, CircS alone, MetS alone, and both CircS and MetS groups was 6.96, 8.77, 7.83, and 10.77%, respectively. The AUCs for CircS predicting prevalent and incident LUTS/BPH were higher than those for MetS (0.582 vs. 0.556 for incident LUTS/BPH, *P* < 0.001; 0.574 vs. 0.561 for prevalent LUTS/BPH, *P* < 0.05).

**Conclusions:** The CircS predicts both incident and prevalent LUTS/BPH better than MetS.

## Introduction

Benign prostatic hyperplasia (BPH) is ubiquitous in aging men (1). The enlarged prostate can subsequently lead to bladder outlet obstruction at the level of the bladder neck and further lower urinary tract symptoms (LUTS) (2). The etiologies of LUTS can be attributed to a variety of conditions; however, it was recognized that BPH is the leading cause (3). Previous studies have disclosed that aging men have a high prevalence of BPH (4). This number can be highly reached to >80% among men older than 80 years (5). Patients with LUTS/BPH will undergo depression (6), reduced sleep duration (4), etc., placing heavy adverse impact on their quality of life. Exploring its risk factors seems requisite and facilitates early prevention.

In the previous literatures, several risk factors comprising age, cigarette consumption, alcohol consumption, low testosterone, etc., were identified (1, 7). Among these risk factors, it is well-documented that metabolic syndrome (MetS) enhances the development and progression of LUTS/BPH *via* activating prostate inflammatory signal pathways (8, 9). MetS is defined as a risk cluster including central obesity, dyslipidemia, hypertension, and hyperglycemia (10). In modern society, the prevalence of metabolic diseases surges. According to the report from Ge et al., the prevalence of MetS is 33.83% in China (11). However, the patients with MetS are usually comorbid with the reduced sleep duration, depression, and cognitive disorder, etc. (12). It indicates that MetS may not be a reasonable cluster, and some underlying mechanisms behind MetS may play a more central role (12). Although some hypotheses such as insulin resistance and central obesity-related inflammation are proposed, little consensus exists. This also hinders the intervention and prevention for LUTS/BPH.

Recently, the concept of circadian syndrome (CircS) is proposed as a common underlying etiology to explain MetS and its comorbidities including depression and reduced sleep duration, which are also commonly comorbid with LUTS/BPH (4, 12). Currently, mounting evidence shows that individuals with common modern lifestyles (more foods and calories intake, less sleeping time and exercises, abuse of artificial light, etc.) are exposed to the dysfunction of circadian rhythms (12). It will negatively affect men and subsequently lead to obesity, sleep disturbance, depression, diabetes, hypertension, etc. (13). Our previous studies also disclosed that men with depression and/or insomnia have higher odds of suffering LUTS/BPH (4). Hence, it indicates that CircS, more than MetS, should be considered as a novel risk cluster for patients with LUTS/BPH, but currently, no study has yet explored the relationship between CircS and LUTS/BPH and compared the predictive values between CircS, MetS, and LUTS/BPH. To investigate whether CircS plays more pivotal role for LUTS/BPH or not, we used the dataset from the China Health and Retirement Longitudinal Study (CHARLS) and performed this study.

## Materials and Methods

### Data Source and Analytical Sample

**Figure 1:**
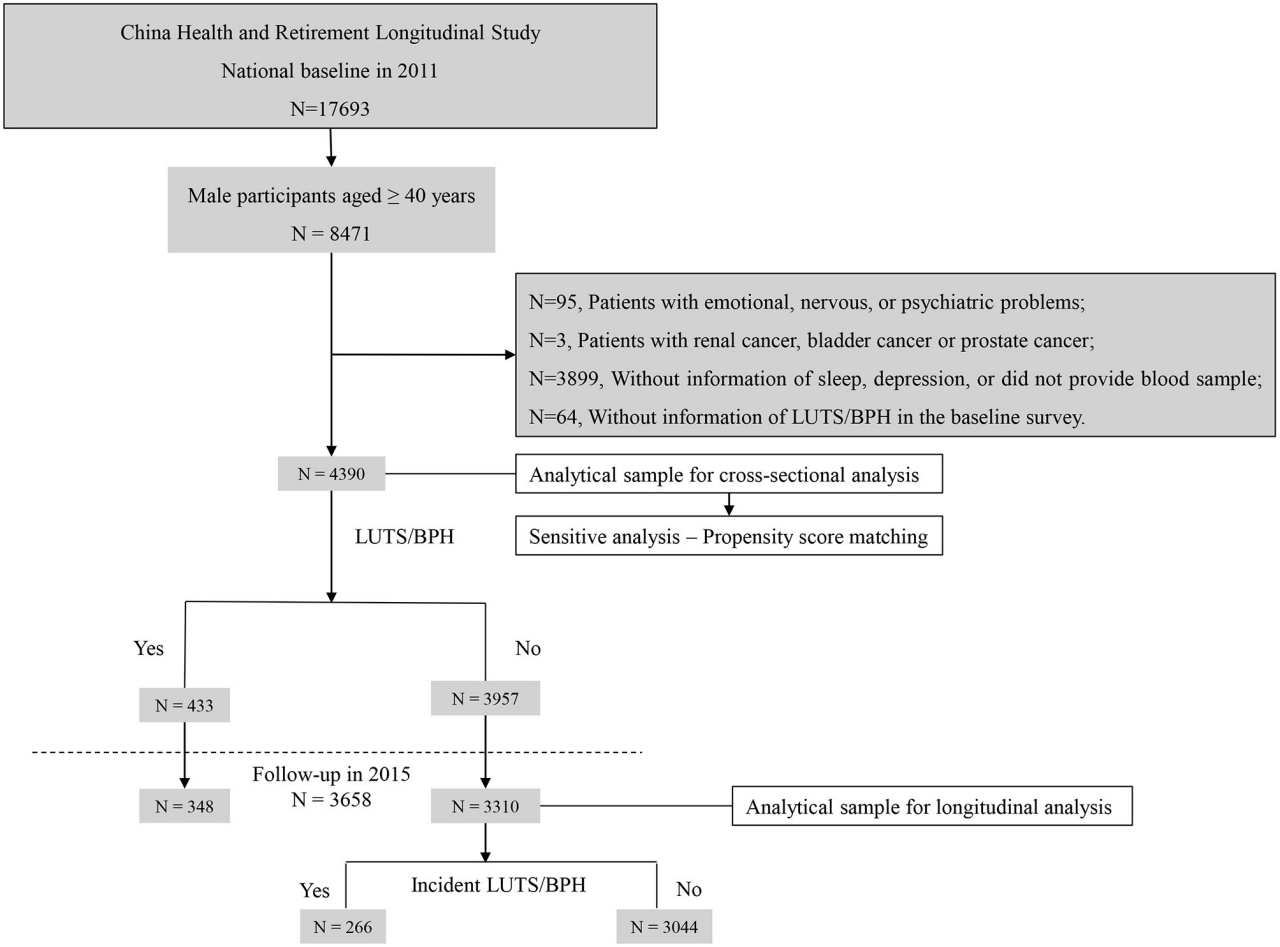
Sample flow chart. N, number; LUTS/BPH, lower urinary tract symptoms suggestive of benign prostate hyperplasia.

In this study, the dataset from the CHARLS was analyzed. CHARLS was a longitudinal project initiated in 2011 and followed up every 2 years in China (14). Adults aged ≥ 45 years in 28 provinces were sampled and investigated. To test the association between the CircS and LUTS/BPH and compare the predictive value between CircS and MetS, the 2011 baseline and 2015 follow-up data were employed to construct the retrospective cohort. In the baseline survey, 8,471 men participants were recruited. After cleansing the dataset, 4,390 men were enrolled into analyses in the baseline survey. Of them, 3,658 men were followed up until 2015 (Figure 1).

### Measurements of Variables: CircS and LUTS/BPH

The concept of CircS was first proposed by Zimmet et al. in 2019 based on the concept of MetS and then defined with a cutoff value by Shi et al. (12, 15). In the definition of CircS, another two risk factors, which were depression and reduced sleep duration, were clustered with the components of MetS including abdominal obesity, hypertension, hyperglycemia, high triglycerides, and low high-density lipoprotein (HDL) cholesterol. In this study, abdominal obesity (waistline ≥ 85 cm), hypertension (systolic pressure ≥ 130 or diastolic pressure ≥ 85 mmHg or drug treatment for hypertension), elevated glucose (≥100 mg dl^−1^ or drug treatment for elevated glucose), high triglycerides (≥150 dl^−1^ or drug treatment for high triglycerides), and low HDL cholesterol (<40 mg dl^−1^ or drug treatment for low HDL cholesterol) were defined according to the previous studies (10). The specific cutoff values of the seven components in CircS and MetS were presented in Supplementary Table 1. According to one previous study, the cutoff for MetS was set as ≥3 components, while for CircS, the cutoff was ≥4 components (15).

The participants were asked to remain fasting in the night, and the researchers collected their venous blood in the next morning. The venous blood was then centrifuged to separate the plasma and stored at −80°C for further determining the triglycerides, glucose, C-reactive protein, uric acid, creatinine, HDL cholesterol, and hemoglobin using enzymatic colorimetric tests. In the morning, when the participants remained seated and quiet for a while, the blood pressure was then measured for three times. These three measurements were recorded, and their mean was calculated to diagnose hypertension. Waist circumference was determined using a measuring tape at the navel level when the participants stood still.

Reduced sleep duration was defined as sleeping <6 h. Depression was assessed using the Epidemiological Studies Depression Scale-10 (CESD-10). Scores ≥ 10 were diagnosed as depression (16). Furthermore, all the relevant drug treatment histories for elevated glucose, low HDL cholesterol, high triglycerides, and hypertension were recorded through a personal face-to-face interview. The collection of blood samples and further tests for blood biomarkers were all performed by professional medical staffs, and the personal face-to-face interviews were finished by at least two staffs together.

Generally, BPH is a term based on histological alterations. Several men with BPH are asymptomatic who, therefore, do not undergo further examinations, diagnosis, and treatments. Enlarged prostate can compress the urethra and then lead to frequent voiding, nocturia, urine retention, etc., namely, LUTS. This condition severely affects men only when it correlates with LUTS. In light of this circumstance, “LUTS attributed to BPH” was abbreviated as LUTS/BPH and used in the previous studies (17). In CHARLS, participants were interviewed if they have ever been diagnosed with a prostate illness (excluding prostatic cancer). Related LUTS, including frequent voiding, nocturia, and urine retention, were interpreted to them. If “Yes,” the participants were defined as suffering LUTS/BPH. This method for diagnosing LUTS/BPH was also adopted in the previous studies (6, 18).

### Covariates

In this study, the following covariates were summarized: age, marital status [married/cohabitated, others (divorced, widowed, never married, and separated)], education, smoking (current smoker, non-smoker, and exsmoker), alcohol consumption (drinker or non-drinker), uric acid, anemia (<120 g/l), stroke (yes or no), and chronic kidney disease (CKD, yes or no). The estimated glomerular filtration rate (eGFR) was calculated to evaluate the kidney function using CKD-EPI creatinine equation according to one previous study (15). The cutoff for diagnosing CKD was set as <60 ml min^−1^/1.73 m^2^ (15).

### Statistical Analyses

In this study, continuous variables with normal distribution were displayed as mean ± SD and those with non-normal distribution were showed as median (25–75% quantiles). Furthermore, categorical data were summarized as proportions (%). Differences within the groups were tested using one-way ANOVA, Kruskal–Wallis, or chi-square test according to the data type and distribution. Furthermore, multiple comparisons between groups were adjusted by the Bonferroni method.

To test the cross-sectional relationship between CircS, MetS, and LUTS/BPH, logistic regression was used. Moreover, in this part, propensity score matching (PSM) using MatchIt package was employed for sensitivity analyses. The 1:1 nearest neighbor matching was used to balance the differences between covariates, and then, ORs were calculated. Furthermore, we excluded the participants who had LUTS/BPH at the baseline to test the longitudinal relationship between CircS, MetS, and LUTS/BPH. *P* < 0.05 (two-sided) were seen as significant in statistics.

To compare the predictive power using the number of CircS and MetS components, receiver operating characteristic (ROC) curve analysis was used. PROC package was adopted to calculate the AUCs and draw the ROC curve. The DeLong test (one-sided) was employed to compare the difference of area under curves (AUCs). All the statistical analyses were performed using R 3.6.3 (R Foundation for Statistical Computing, Vienna, Austria).

## Results

### Characteristics of the Participants Attending the Baseline Survey

**Figure 2:**
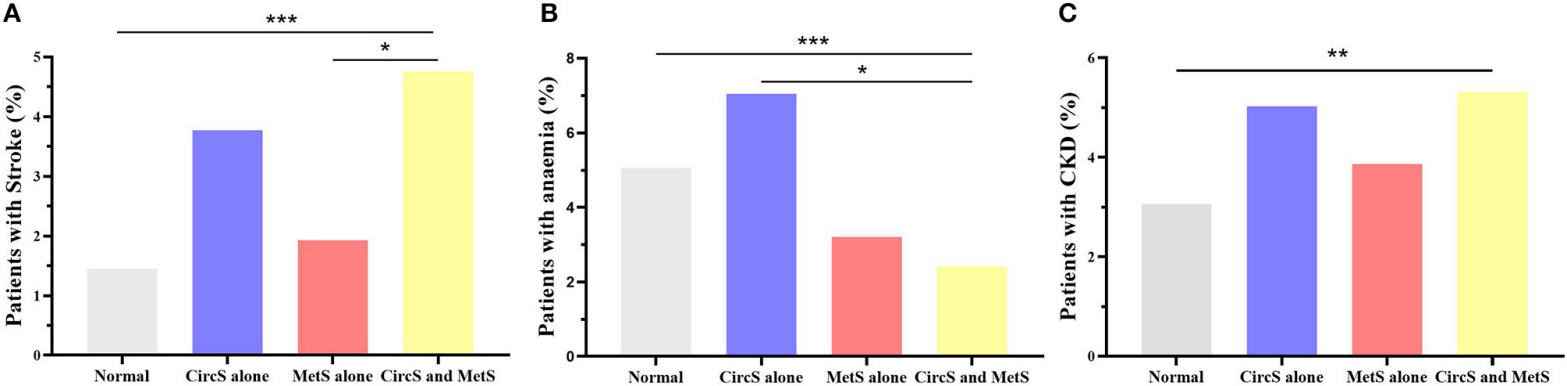
Disparities in the prevalence of stroke, anemia, and chronic kidney disease in the four groups. **(A)** showed the disparities in the prevalence of stroke. **(B)** displayed the disparities in the prevalence of anemia. **(C)** presented the disparities in the prevalence of chronic kidney diseases. Fisher's precision probability test was adopted to test the differences. Furthermore, multiple comparisons between the four groups were adjusted by the Bonferroni method. MetS, metabolic syndrome; CircS, circadian syndrome; CKD, chronic kidney disease. *P < 0.05; **P < 0.01; ***P < 0.001.

A total of 4,390 participants attending the CHARLS baseline survey in 2011 were enrolled (Table 1). The mean age of the enrolled participants was 60.23 (SD = 9.33). The participants were divided into the following four groups: normal, CircS alone, MetS alone, and both CircS and MetS. It was noted that CircS alone group had higher prevalence of stroke, anemia, and CKD than the MetS alone group (3.77 vs. 1.94%; 7.05 vs. 3.21%; and 5.03 vs. 3.87%, Figure 2). Moreover, all the CircS alone group had reduced sleep duration and depression. None of the participants in MetS alone group had depression, and of them, only 3.16% had reduced sleep duration.

**Table 1:** Characteristics of the participants attending the baseline survey.

**Characteristics**	**Total** ***N* = 4,390**	**Normal** ***N* = 2,547**	**CircS alone** ***N* = 159**	**MetS alone** ***N* = 570**	**Both CircS and MetS** ***N* = 1,114**	***P***
Age (years)	60.23 (9.33)	60.35 (9.42)	62.74 (9.48)	59.90 (9.58)	59.76 (8.90)	<0.01
Marital status						<0.001
Married/cohabitated	3,862 (87.97%)	2,243 (88.06%)	115 (72.33%)	515 (90.35%)	989 (88.78%)	
Others	528 (12.03%)	304 (11.94%)	44 (27.67%)	55 (9.65%)	125 (11.22%)	
Educational levels						<0.001
Illiterate	1,471 (33.52%)	919 (36.10%)	80 (50.31%)	161 (28.30%)	311 (27.92%)	
Elementary school	1,197 (27.28%)	707 (27.77%)	42 (26.42%)	138 (24.25%)	310 (27.83%)	
Middle school	1,137 (25.91%)	615 (24.16%)	31 (19.50%)	160 (28.12%)	331 (29.71%)	
High school	374 (8.52%)	217 (8.52%)	5 (3.14%)	63 (11.07%)	89 (7.99%)	
College and above	209 (4.76%)	88 (3.46%)	1 (0.63%)	47 (8.26%)	73 (6.55%)	
Smoking						<0.001
Non-smoker	1,081 (24.62%)	578 (22.69%)	28 (17.61%)	164 (28.77%)	311 (27.92%)	
Ex-smoker	753 (17.15%)	378 (14.84%)	20 (12.58%)	103 (18.07%)	252 (22.62%)	
Current smoker	2,556 (58.22%)	1,591 (62.47%)	111 (69.81%)	303 (53.16%)	551 (49.46%)	
Alcohol consumption						<0.05
Yes	2,468 (56.22%)	1,459 (57.28%)	85 (53.46%)	335 (58.77%)	589 (52.87%)	
No	1,922 (43.78%)	1,088 (42.72%)	74 (46.54%)	235 (41.23%)	525 (47.13%)	
High-sensitivity CRP (≥10 mg. dL^−1^)	1.09 (0.57–2.30)	0.90 (0.50–1.94)	0.86 (0.51–2.57)	1.30 (0.69–2.38)	1.47 (0.75–3.02)	0.2435
Uric acid (mg. dL^−1^)	4.96 (1.27)	4.79 (1.21)	4.78 (1.11)	5.10 (1.29)	5.31 (1.36)	<0.001
BMI (Kg. m^−2^)	23.01 (3.71)	21.59 (2.96)	21.80 (4.07)	24.75 (3.32)	25.58 (3.65)	<0.001
Stroke	107 (2.44%)	37 (1.46%)	6 (3.77%)	11 (1.94%)	53 (4.77%)	<0.001
Anemia	181 (4.23%)	126 (5.06%)	11 (7.05%)	18 (3.21%)	26 (2.42%)	<0.01
CKD	166 (3.80%)	78 (3.06%)	8 (5.03%)	22 (3.87%)	58 (5.32%)	<0.05
Abdominal obesity	2,042 (46.51%)	589 (23.13%)	45 (28.30%)	441 (77.37%)	967 (86.80%)	<0.001
Reduced sleep duration	1,197 (27.28%)	640 (25.14%)	159 (100.00%)	18 (3.16%)	380 (34.11%)	<0.001
Depression	1,287 (30.55%)	692 (28.40%)	159 (100.00%)	0 (0.00%)	436 (39.14%)	<0.001
Systolic BP (mmHg)	130.90 (22.53)	125.38 (20.25)	136.33 (22.23)	137.14 (19.22)	139.60 (25.25)	<0.001
Diastolic BP (mmHg)	76.30 (12.49)	73.23 (11.73)	77.14 (12.70)	80.14 (11.88)	81.26 (12.30)	<0.001
Hypertension	1,685 (38.38)	603 (23.67)	80 (50.31)	288 (50.53)	714 (64.09)	<0.001
Elevated plasma glucose	2,584 (58.86)	1,069 (41.97)	109 (68.55)	433 (75.96)	973 (87.34)	<0.001
Elevated serum triglycerides	1,191 (27.13)	144 (5.65)	18 (11.32)	221 (38.77)	808 (72.53)	<0.001
Elevated blood pressure	2,319 (52.82)	897 (35.22)	110 (69.18)	418 (73.33)	894 (80.25)	<0.001
Reduced serum HDL	1,346 (30.66)	246 (9.66)	36 (22.64)	242 (42.46)	822 (73.79)	<0.001
Circadian syndrome	1,273 (30.23)	0 (0.00)	159 (100.00)	0 (0.00)	1,114 (100.00)	<0.001
Metabolic syndrome	1,684 (38.36)	0 (0.00)	0 (0.00)	570 (100.00)	1,114 (100.00)	<0.001
LUTS/BPH cases in 2011	433 (9.86)	207 (8.13)	22 (13.84)	54 (9.47)	150 (13.46)	<0.001
LUTS/BPH cases in 2015	266 (8.04)	138 (6.96)	10 (8.77)	34 (7.83)	84 (10.77)	<0.05

*MetS, metabolic syndrome; CircS, circadian syndrome; LUTS/BPH, lower urinary tract symptoms suggestive of benign prostatic hyperplasia; CRP, C-reactive protein; BMI, body mass index; HDL, high-density lipoprotein; CKD, chronic kidney disease*.

### The Cross-Sectional Association Between MetS, CircS, and Prevalent LUTS/BPH

**Figure 3:**
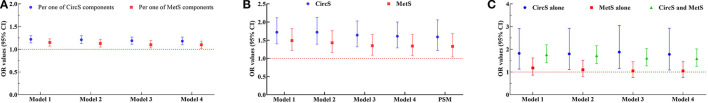
The cross-sectional association between metabolic syndrome (MetS), circadian syndrome (CircS), and prevalent LUTS/BPH. **(A)** Odds ratios (ORs) of the CircS and MetS groups varied with the numbers of their components; **(B)** ORs of CircS and MetS groups; **(C)** ORs of the CircS alone, MetS alone, and both CircS and MetS groups. ORs were calculated using logistic regression. Model 1 was performed using univariate logistic regression. Model 2 was adjusted for age, education, marital status, and uric acid in blood. Model 3 was further adjusted for smoking and alcohol consumption. Model 4 was then further adjusted for anemia, stroke, and CKD. Propensity scores matching (PSM) was performed using MatchIt package in R 3.6.3. MetS, metabolic syndrome; CircS, circadian syndrome; PSM, propensity scores matching; OR, odds ratios; CI, confidence interval.

It was indicated that both CircS and MetS correlated positively with prevalent LUTS/BPH. In Figure 3A, the numbers for CircS and MetS components were set as independent variables. All the ORs for CircS were higher than those for MetS in all the four models, suggesting that patients with CircS had higher risks for prevalent LUTS/BPH than patients with MetS. In Figure 3B, after adjusted for all the covariates (Model 4), the OR value for CircS was 1.61 (95% CI 1.29–2.00, *P* < 0.001), while that for MetS was 1.34 (1.08–1.66, *P* < 0.01). To balance the differences between covariates, PSM analysis was performed. After PSM, all the mean differences were <10%, indicating a good attenuation of the disparities between the groups. It was revealed that the CircS group had 1.59 times higher risks for prevalent LUTS/BPH (95% CI 1.22–2.06, *P* < 0.001) than their counterparts and that the ORs for the MetS group were 1.33 (1.04–1.68, *P* < 0.05). To detect the risks for patients who had both CircS and MetS, this subgroup was separated and subjected to further analyses (Figure 3C). After adjusted for all the covariates (Model 4), it was revealed that patients having both CircS and MetS had 1.59 times higher risks for prevalent LUTS/BPH than the normal participants (95% CI 1.25–2.02, *P* < 0.001), which was lower than that for the CircS alone group (ORs: 1.59 vs. 1.78), but higher than the MetS alone group (ORs: 1.59 vs. 1.05).

### The Longitudinal Association Between MetS, CircS, and Incident LUTS/BPH

The patients with LUTS/BPH in 2011 were excluded, and the rest of the participants were followed to 2015. ORs for CircS were higher than those for MetS in all four models. In Model 4, it was disclosed that having CircS was linked to 150% increased risks of developing LUTS/BPH, while the increased risks for MetS were 132% (Table 2).

**Table 2:** The longitudinal association between MetS and CircS status and incident LUTS/BPH (2011–2015).

**Models**	**MetS**		**CircS**	
	**Per one of MetS components [ORs (95% CI)]**	**MetS [ORs (95% CI)]**	**Per one of CircS components [ORs (95% CI)]**	**CircS [ORs (95% CI)]**
Model 1	1.17 (1.07–1.27)^***^	1.42 (1.10–1.82)^**^	1.17 (1.08–1.27)^***^	1.53 (1.18–2.00)^**^
Model 2	1.16 (1.06–1.27)^***^	1.37 (1.06–1.76)^*^	1.18 (1.09–1.28)^***^	1.57 (1.20–2.05)^***^
Model 3	1.15 (1.05–1.26)^**^	1.31 (1.01–1.70)^*^	1.17 (1.08–1.28)^***^	1.54 (1.18–2.03)^**^
Model 4	1.14 (1.04–1.25)^**^	1.32 (1.01–1.72)^*^	1.17 (1.07–1.27)^***^	1.50 (1.14–1.98)^**^

*Model 1 was performed using univariate logistic regression and adjusted for no covariates. Model 2 was adjusted for age, education, marital status, and uric acid in blood. Model 3 was further adjusted for smoking and alcohol consumption. Model 4 was then further adjusted for anemia, stroke, and chronic kidney disease.*

*MetS, metabolic syndrome; CircS, circadian syndrome; OR, odds ratios.*

* *P < 0.05.*

** *P < 0.01.*

*** *P < 0.001*.

### The Predictive Power of CircS and MetS

**Figure 4:**
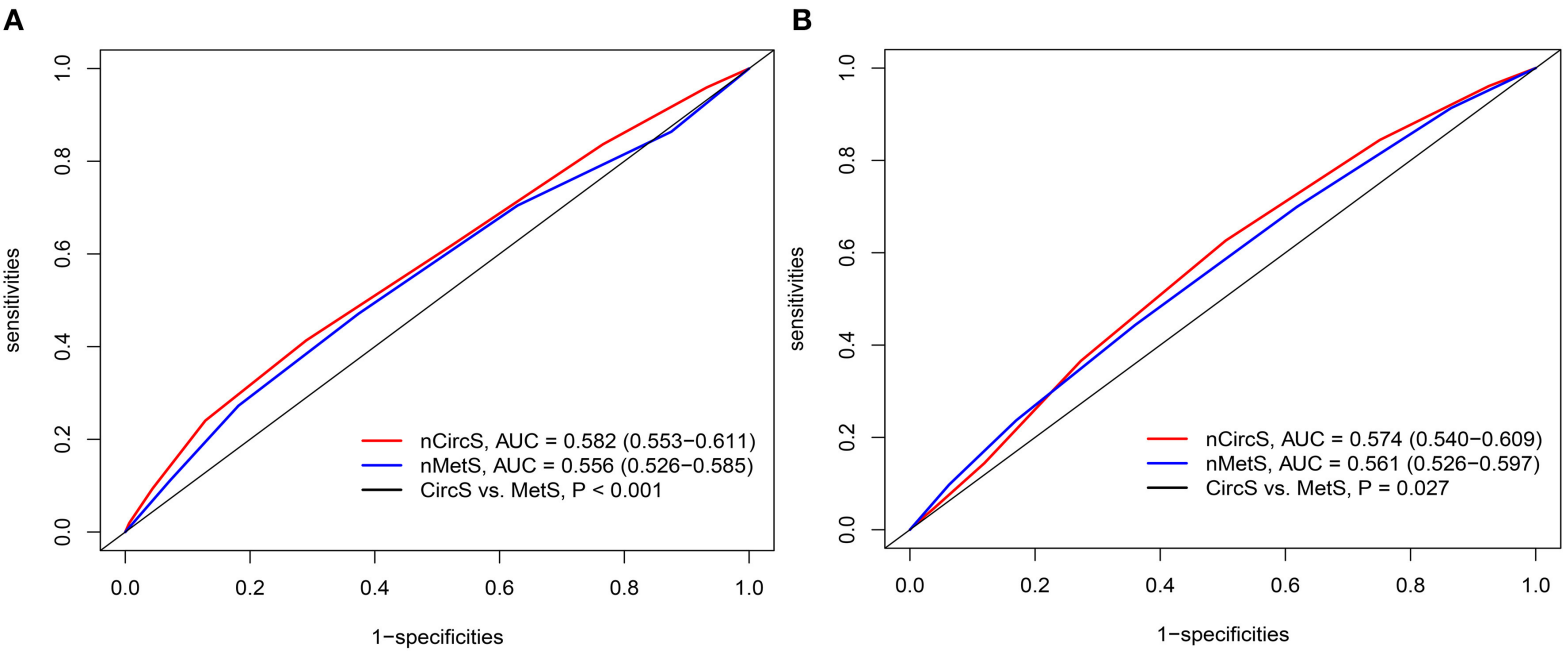
The receiver operating characteristic curve (ROC) curves for prevalent and incident LUTS/BPH. **(A)** ROCs for the prevalent LUTS/BPH in 2011. **(B)** ROCs for the incident LUTS/BPH (2011–2015). PROC package in R 3.6.3 was adopted to perform ROC analyses. DeLong test was employed to compare the AUCs for CircS and MetS. The number of CircS and MetS components were set as independent variables. nMetS, number of metabolic syndrome components; nCircS, number of circadian syndrome components; ROC, receiver operating characteristic curve; AUC, area under curve.

To compare the predictive power of CircS and MetS, the ROCs were drawn (Figure 4). For the prevalent LUTS/BPH, the AUCs for CircS and MetS were 0.582 (0.553–0.611) and 0.556 (0.526–0.585), respectively. For the incident LUTS/BPH, the AUCs for CircS were 0.574 (0.540–0.609), which was higher than that for MetS [AUC = 0.561 (0.526–0.597), *P* < 0.05]. It indicated that CircS had better predictive powers than MetS both for prevalent and incident LUTS/BPH.

## Discussion

In this nationally longitudinal study targeting the elderly, we found that CircS was positively correlated with LUTS/BPH and patients with CircS were under significantly higher risks of both prevalent and incident LUTS/BPH than those with MetS. The CircS was a better predictor for LUTS/BPH than MetS.

This association between MetS and LUTS/BPH was in line with previous findings (19–22). Xiong et al. found that men with MetS had 1.47 times of LUTS/BPH than the healthy counterparts in Chinese aging men (19). Cosimo et al. also disclosed that MetS increased the risk of moderate/severe nocturia in patients with LUTS/BPH (22). However, as far as we knew that CircS was first revealed as a risk cluster for LUTS/BPH in this study. Previous surveys have revealed that patients with LUTS/BPH had higher prevalence of depression and shorter sleep duration (4, 23, 24); however, depression, sleep disturbance, and the components of MetS were usually considered separately and evaluated hardly at the same time. No one common etiology for explaining these risk factors simultaneously was proposed. In this study, depression and reduced sleep duration were assessed, and the further constructed risk cluster displayed better predictive power, indicating that the circadian dysfunction may act as a more central role in the mechanism of developing LUTS/BPH.

The regulation of circadian rhythm in organisms depends on circadian clock genes (13). It was reported that aggressive prostate cancer was closely linked with three core circadian clock genes, *ARNTL, NPAS2*, and *RORA* (25). However, how the circadian clock regulates the cells in the prostate remains unclear. According to the previous studies, it was postulated that testosterone might be the mediating role linking CircS to BPH. In some clinical studies, men with reduced sleep duration or MetS or depression were observed to have decreased testosterone (8). In the rat prostate, Kawamura et al. found that the expression of two core circadian clock genes, *Bmal1* and *Per2*, decreased with the reduced testosterone (26). Moreover, as reported by Li et al., the decreased expression of *Per2* can inhibit apoptosis (27), which then may lead to the occurrence of BPH (26). This hypothesis still needs further evidence to demonstrate, which is what we plan to do in future studies.

This study also revealed that MetS alone group was not associated with LUTS/BPH, while CircS alone and both CircS and MetS were associated with LUTS/BPH. This finding highlighted the importance of incorporating depression and reduced sleep duration into a novel risk cluster. Furthermore, 3.62% participants who had CircS alone were found with higher risks than the MetS alone counterparts. This subgroup cannot be labeled as MetS and then might be ignored by doctors. Considering the low cost of evaluating the mental status and sleep quality, depression and insomnia should be noted in clinical settings, which may facilitate early prevention.

Moreover, it should also be noted that the CircS alone group had different clinical characteristics. It was revealed that higher weight gain was linked to higher risks of developing LUTS/BPH (8). However, the body mass index (BMI) in CircS alone group was relatively low, but the prevalence of stroke, CKD, and anemia was higher than that in MetS group. It indicates that circadian dysfunction may play a more central underlying role in this subgroup, rather than obesity.

In this study, although a national longitudinal survey was used to investigate the association between CircS and LUTS/BPH, some limitations still should be noted. First, the follow-up duration was short. A 4-year follow-up duration may not be long enough to find more obvious effects from CircS. Furthermore, a simplified method to define LUTS/BPH according to method of Zhang was adopted (18). The definition of LUTS/BPH was mainly based on self-report as opposed to more objective tests such as prostatic ultrasonography, which may bias the diagnoses. In future studies, more objective measurements should be adopted.

In conclusion, our study shows that the CircS may act as a more central role in developing LUTS/BPH. The CircS predicts both incident and prevalent LUTS/BPH better than MetS. In clinical settings, men who cannot be diagnosed as MetS still should beware of CircS.

## Conclusions

The CircS predicts both incident and prevalent LUTS/BPH better than MetS.

## Data Availability Statement

The datasets presented in this study can be found in online repositories. The names of the repository/repositories and accession number(s) can be found below: http://charls.pku.edu.cn/.

## Ethics Statement

The studies involving human participants were reviewed and approved by Ethics Committee of Peking University. The patients/participants provided their written informed consent to participate in this study.

## Author Contributions

YX and FZ performed the data analyses and wrote the manuscript. CW, YZ, and FQ revised the manuscript. JY participated in the study design and helped draft the manuscript. All authors contributed to the article and approved the submitted version.

## Funding

This work was supported by the Natural Science Foundation of China (81871147 and 82071639) and the Chengdu Science and Technology Program (2019-YFYF-00087-SN).

## Conflict of Interest

The authors declare that the research was conducted in the absence of any commercial or financial relationships that could be construed as a potential conflict of interest.

## Publisher's Note

All claims expressed in this article are solely those of the authors and do not necessarily represent those of their affiliated organizations, or those of the publisher, the editors and the reviewers. Any product that may be evaluated in this article, or claim that may be made by its manufacturer, is not guaranteed or endorsed by the publisher.
