# Supplementary material for: The Circadian Syndrome Predicts Lower Urinary Tract Symptoms Suggestive of Benign Prostatic Hyperplasia Better Than Metabolic Syndrome in Aging Males: A 4-Year Follow-Up Study
Source: Front Med (Lausanne). 2021 Sep 21;8:715830. doi: 10.3389/fmed.2021.715830 (PMC8490706; doi:10.3389/fmed.2021.715830)
Supplement: Supplementary file 1 [file Table_1.DOCX]

| **Supplemental Table 1: The diagnostic criteria and cutoff values for MetS and CircS in aging males** | | | | |
| --- | --- | --- | --- | --- |
| **Components** | **Cutoff values** | **Medical histories** | **MetS** | **CircS** |
| **Reduced sleep duration** | Sleeping time < 6 hours | NA | × | √ |
| **Depression** | CESD-10 scores ≥ 10 | NA | × | √ |
| **Abdominal obesity** | waistline ≥ 85 cm | NA | √ | √ |
| **Hypertension** | SP ≥ 130 or DP ≥ 85 mmHg | drug treatment for hypertension | √ | √ |
| **Elevated glucose** | ≥ 100 mg dL-1 | drug treatment for elevated glucose | √ | √ |
| **Elevated TG** | ≥ 150 mg dL-1 | drug treatment for high TG | √ | √ |
| **Reduced HDL** | < 40 mg dL-1 | drug treatment for low HDL cholesterol | √ | √ |
| **Diagnostic criteria** | NA | NA | ≥ three components | ≥ four components |

**Notes**: NA: not applicable. CESD-10: Epidemiological Studies Depression Scale-10. SP: systolic pressure. DP: diastolic pressure. TG: triglycerides. HDL: high-density lipoprotein. **Men with medical histories or values meeting the cutoff are seen as positive in this item**.
